# Modeling SARS-CoV-2 nucleotide mutations as a stochastic process

**DOI:** 10.1371/journal.pone.0284874

**Published:** 2023-04-28

**Authors:** Maverick Lim Kai Rong, Ercan Engin Kuruoglu, Wai Kin Victor Chan

**Affiliations:** Tsinghua-Berkeley Shenzhen Institute, Tsinghua University, Shenzhen, China; University of Bologna / Romagna Local Health Authority, ITALY

## Abstract

This study analyzes the SARS-CoV-2 genome sequence mutations by modeling its nucleotide mutations as a stochastic process in both the time-series and spatial domain of the gene sequence. In the time-series model, a Markov Chain embedded Poisson random process characterizes the mutation rate matrix, while the spatial gene sequence model delineates the distribution of mutation inter-occurrence distances. Our experiment focuses on five key variants of concern that had become a global concern due to their high transmissibility and virulence. The time-series results reveal distinct asymmetries in mutation rate and propensities among different nucleotides and across different strains, with a mean mutation rate of approximately 2 mutations per month. In particular, our spatial gene sequence results reveal some novel biological insights on the characteristic distribution of mutation inter-occurrence distances, which display a notable pattern similar to other natural diseases. Our findings contribute interesting insights to the underlying biological mechanism of SARS-CoV-2 mutations, bringing us one step closer to improving the accuracy of existing mutation prediction models. This research could also potentially pave the way for future work in adopting similar spatial random process models and advanced spatial pattern recognition algorithms in order to characterize mutations on other different kinds of virus families.

## 1 Introduction

The COVID-19 pandemic has wreaked havoc across all corners of the globe ever since its initial emergence at the end of 2019. As of this writing, there have been more than 200 million recorded infections, resulting in over 5 million deaths [1]. Scientists all around the world have worked tirelessly to combat the SARS-CoV-2 virus which caused this deadly disease. Through careful genome sequencing, researchers seek to gain a better understanding of this lethal foe.

Accurate estimates of virus mutation rates play an integral role in understanding the evolution of viruses and the tactics to combat them. This is of critical importance and urgency especially during widespread outbreaks, such as the COVID-19 pandemic that still ravages parts of the world even over two years since the outbreak. There remains a constant race between the production of effective vaccines versus the mutation of new virus strains that could threaten to render existing vaccines obsolete.

The media has increasingly highlighted evidence of an alarming increase in the number of “breakthrough cases” [2], whereby individuals are still becoming reinfected by the virus despite having already been vaccinated against it. Thus, it has become increasingly imperative to predict when and where the next mutation would occur ahead of the actual mutation. This will allow vaccine manufacturers to remain a step ahead of the virus, enabling them to preemptively prepare for quick adaption of the vaccine production process, potentially saving countless lives.

With the constant emergence of new dominant strains, we require regular vaccine shots to boost our immunity, much like the seasonal flu. For this purpose, this study seeks to predict when and where the virus could mutate next, which can then be passed to virologists for further analysis.

**Fig 1:**
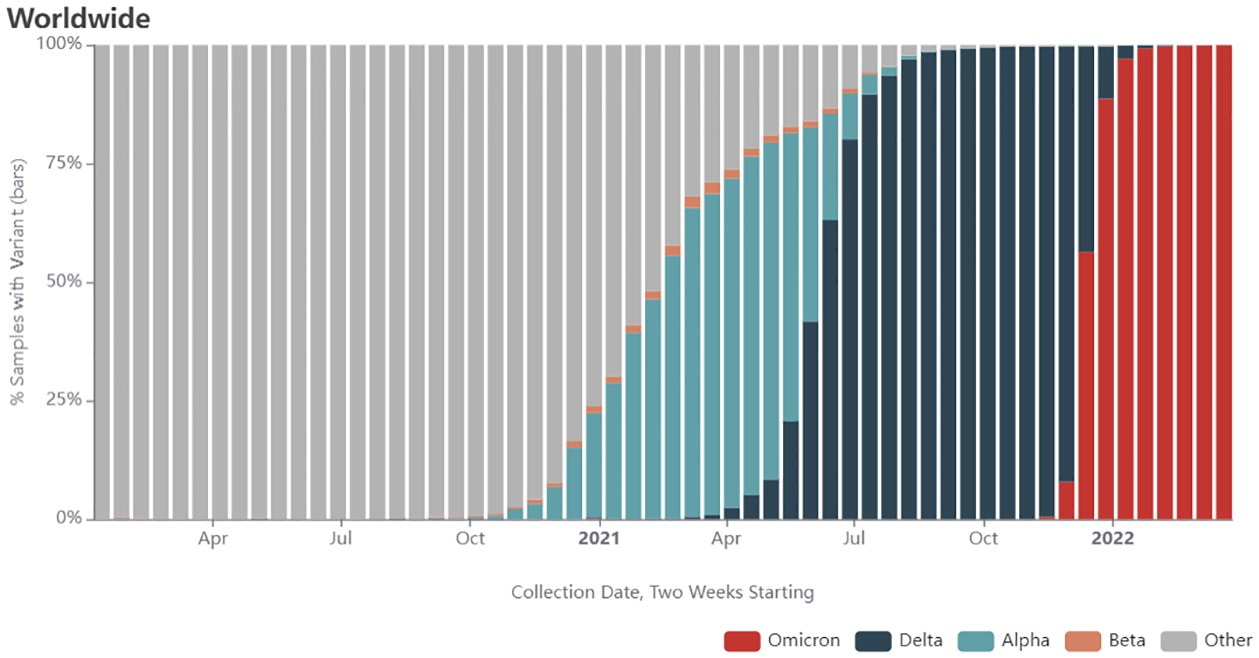
SARS-CoV-2 variants distribution [3].

Our study focuses on five key variants of concern (VOCs) that have become a global concern due to high transmissibility and virulence. These are namely the B.1.1.7 (Alpha), B.1.351 (Beta), P.1 (Gamma), B.1.617.2 (Delta) and B.1.1.529 (Omicron) variants which had become the globally dominant strains over different periods of the pandemic, typically lasting over several months before the emergence of the next dominant VOC (Fig 1).

As such, there is potential for COVID-19 to eventually become endemic in the global population, which means that the SARS-CoV-2 virus could be here to stay and coexist with humanity. Much like with the flu, the best way to combat this is to require a regular preventive vaccine shot to continuously bolster our immunity against new strains of the virus. In this vein, the more critical questions would thus be—How often would we need this booster shot; and how often would we have to update the vaccine sequences? This study seeks to provide an indicative answer to these questions by studying the virus phylodynamics, or how it mutates over time. Through a detailed survey of related works which will be explored in (subsection 1.1), we have noted that while much research has been done on its evolution phylogeny, few studies have delved specifically into the time dynamics. Therefore, our study seeks to close this research gap by carrying out a comprehensive time-series analysis of the virus mutation dynamics by modeling it as a stochastic process.

We also go one step further beyond the time-series modelling by conducting spatial gene sequence analysis on the virus nucleotide sequences. Such analysis would not only reveal when we expect mutations to occur, but also at what positions they could be found along the genome. In particular, our spatial gene sequence model focuses on identifying patterns in the mutation interoccurrence distances between nucleotides. Research by Muino et al. (2014) [4] has found that cancer mutations exhibit power-law distributions for certain short-range interoccurrence distances. Although this attribute was found to be specific to cancer genomes, there is also potential to uncover a similarly characteristic pattern that could be unique to specific viruses such as SARS-CoV-2. As of this writing, we are not aware of any similar spatial gene sequence study conducted on the SARS-CoV-2 genome to characterize its mutation interoccurrence properties.

The findings from both the time-series and spatial domains could contribute interesting insights to the underlying biological mechanism of SARS-CoV-2 mutations, which brings us one step closer to improving the accuracy of existing mutation prediction models. This information can then be passed on to experts to determine the corresponding virological impact of such predicted mutations. This research could also potentially pave the way for future work such as adopting spatial random process models on advanced spatial pattern recognition algorithms in order to characterize mutations on various kinds of virus families.

### 1.1 Related works

Existing methods of virus mutation estimation models are diverse and often highly complex in nature [5]. A number of mathematical models for cell populations in which mutations are occurring have been studied [6]. We explore a panorama of previous works related to the modeling of virus RNA sequence mutation using a variety of innovative methods. For ease of comparison, we categorize their methods into two types of general approaches, either through a probabilistic/stochastic approach or a pattern recognition-based approach. In this study, our model falls into the former category.

#### 1.1.1 Probabilistic/stochastic approaches

Muiño et al. (2014) [4] studied the spatial gene sequence distribution of mutation in cancer cells and discovered that cancer genomes exhibit power-law interoccurrence distances in the short-range. Although this attribute was found to be specific to cancer genomes, there is also potential to uncover a similarly characteristic pattern that could be unique to specific viruses such as SARS-CoV-2. In this paper, we conduct a similar analysis on the SARS-CoV-2 genome to analyze how the spatial distance between nucleotides influence the mutation rate along the sequence. As with each of the aforementioned works, our study seeks to reveal yet another facet of the mutagenic nature of the virus.

De Maio et al. (2021) [7] studied the mutation rates and selection on synonymous mutations in SARS-CoV-2 by counting and comparing the ratio of observed mutation types for descendants. Their results point to evidence that there exists some selection bias on specific mutation types which heavily favoured mutations from G to U and from C to U by a factor of up to 8 times. However, their study of mutation rates only provides the relative ratio of mutation types without the consideration of the mutation dynamics in the actual time domain. Our study also seeks to characterize the propensities for each type of mutation, but further analyzes their dynamics in the time domain through an embedded Poisson process.

Weinstein et al. (2020) [8] introduced a structured emission distribution (the MuE distribution) that accounts for mutational variability (substitutions and indels) and uses it to construct generative and predictive hierarchical Bayesian models (H-MuE models). The H-MuE models can infer latent representations and features for immune repertoires, predict functional unobserved members of disordered protein families and forecast the evolution of pathogens.

Nie et al. (2020) [9] conducted phylogenetic and phylodynamic analyses of SARS-CoV-2 using the Kimura 3-parameter nucleotide substitution model, which assigns different probabilities for transitions and transversions as shown in (Fig 3). In contrast, our method uses a 12-parameter model by assigning a unique probability for each substitution, allowing more specific characterization of each mutation event. Based on their regression model, the overall evolutionary rate was found to be 9.90 × 10^−4^ substitutions per site per year, which translates to approximately 2.475 substitutions per month assuming the average sequence length of 30,000 nucleotide sites. This closely matches our empirical results of 2.27 as summarized in (section 5) of this paper. However, their results only provide a generalized evolutionary rate, whereas our model goes beyond calculating the mutation rates, we also study the time dynamics with a Markov chain embedded Poisson process as detailed in (section 2).

Hallak et al. (2022) [10] conducted a statistical modeling of SARS-CoV-2 substitution processes as a function of ten explanatory factors based on existing biological literature. By comparing all possible combinations of these ten factors as Generalized Linear Models, they identified the top performing models which can predict the variants that are most likely to occur based on their mutational likelihood. In this study, a Poisson regression model was fitted to the following ten explanatory factors: locus of the site, input nucleotide base (A/C/G/U), input amino acid, input codon, position of the site in the codon (1–3), mature peptide indicator, stem loop indicator, CG pair indicator, right and left neighboring nucleotide.

Our survey of several stochastic approaches revealed that while many existing models have investigated the probabilistic behaviour of mutation patterns, few studies have delved into the time domain to specifically characterize their time dynamics. Although it is very useful to know what type of mutation we can expect, it is more imperative to anticipate when such mutation is most likely to occur, since response time is of paramount importance during an ongoing pandemic. Therefore, this study conducts a time-series analysis to also understand the mutation rates behind the stochastic process.

#### 1.1.2 Pattern recognition-based approaches

Darooneh et al. (2022) [11] proposed a novel text-mining method to estimate the mutability of genomic segments directly from a reference (ancestral) whole genome sequence. The underlying assumption behind this model is that the interactions between neighbouring nucleotides results in clustering of segments, which are analogous to the clustering of words by the grammatical structure rules of natural language. Therefore, their method relies on calculating the importance of genomic segments based on their spatial gene sequence distribution and frequency over the whole genome. The application of this research could potentially aid in the formulation of novel therapeutics, which target the stable conserved parts of the virus in order to retain sustained efficacy even for new variants.

Zheng et al. (2009) [12] propose two metrics to compare DNA and protein sequences based on a Poisson model of word occurrences. Instead of comparing the frequencies of all fixed-length words in two sequences, they consider firstly the probability of ‘generating’ one sequence under the Poisson model estimated from the other; secondly their different expression levels of words. This method provides an in-depth look beyond individual nucleotide mutations, but also analyze the set of codons mutation frequencies.

Nawaz et al. (2021) [13] employed a machine learning algorithm called Sequential Pattern Mining (SPM) on a computer-understandable corpus of COVID-19 genome sequences to analyze whether there are any interesting hidden patterns, revealing frequent patterns of nucleotide bases and their relationships with each other. This is then applied to sequence prediction models to evaluate if nucleotide base(s) can be predicted from previous ones. For mutation analysis in genome sequences, an algorithm is designed to find the locations in the genome sequences where the nucleotide bases are changed and to calculate the mutation rate.

Hie et al. (2021) [14] modeled SARS-CoV-2 virus mutations with viral escape using machine learning algorithms originally developed for human natural language. Escape mutations are those that preserve viral infectivity but cause a virus to look different to the immune system, akin to word changes that preserve a sentence’s grammaticality but change its meaning. By analyzing the semantics (set of codons) together with the syntax (grammatical structure of the RNA), the algorithm can even predict whether mutations would be benign or deleterious in nature.

The aforementioned works employ a myriad of pattern recognition methods which focus on identifying certain spatial gene sequence patterns among mutations along the virus genome sequence. In our work, we instead use a stochastic approach to characterize the distribution of inter-occurrence between mutations. Both of these approaches reveal distinct characteristics of the mutation process and can be used in conjunction to understand the underlying spatial gene sequence behaviour of nucleotide substitutions.

### 1.2 The SARS-CoV-2 genome

**Fig 2:**
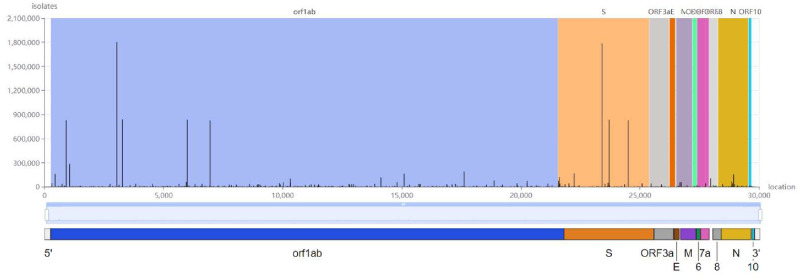
SARS-CoV-2 gene mutation frequency [15].

The SARS-CoV-2 consists of approximately 30,000 nucleotides (labeled as A, G, T, C respectively), each of which could undergo an independent and random mutation during replication (Fig 2). The colors represent the RNA structure components, such as blue for the Open Reading Frame (ORF), orange for the Spike Protein (S) and purple for the cell Membrane (M). From (Fig 2), we can observe that each of these components features sites with higher frequency of mutation, given by bar heights which represent the number of isolates which have detected the corresponding mutations. The significance of this observation suggests that even though the mutation process is random, in the long run there may be selective evolutionary pressures which result in specific types of mutations being preferentially favored over others. Therefore, understanding the underlying mechanism behind this pattern constitutes the main impetus behind this research.

## 2 Methods

### 2.1 A continuous Markov process model for time series analysis

**Fig 3:**
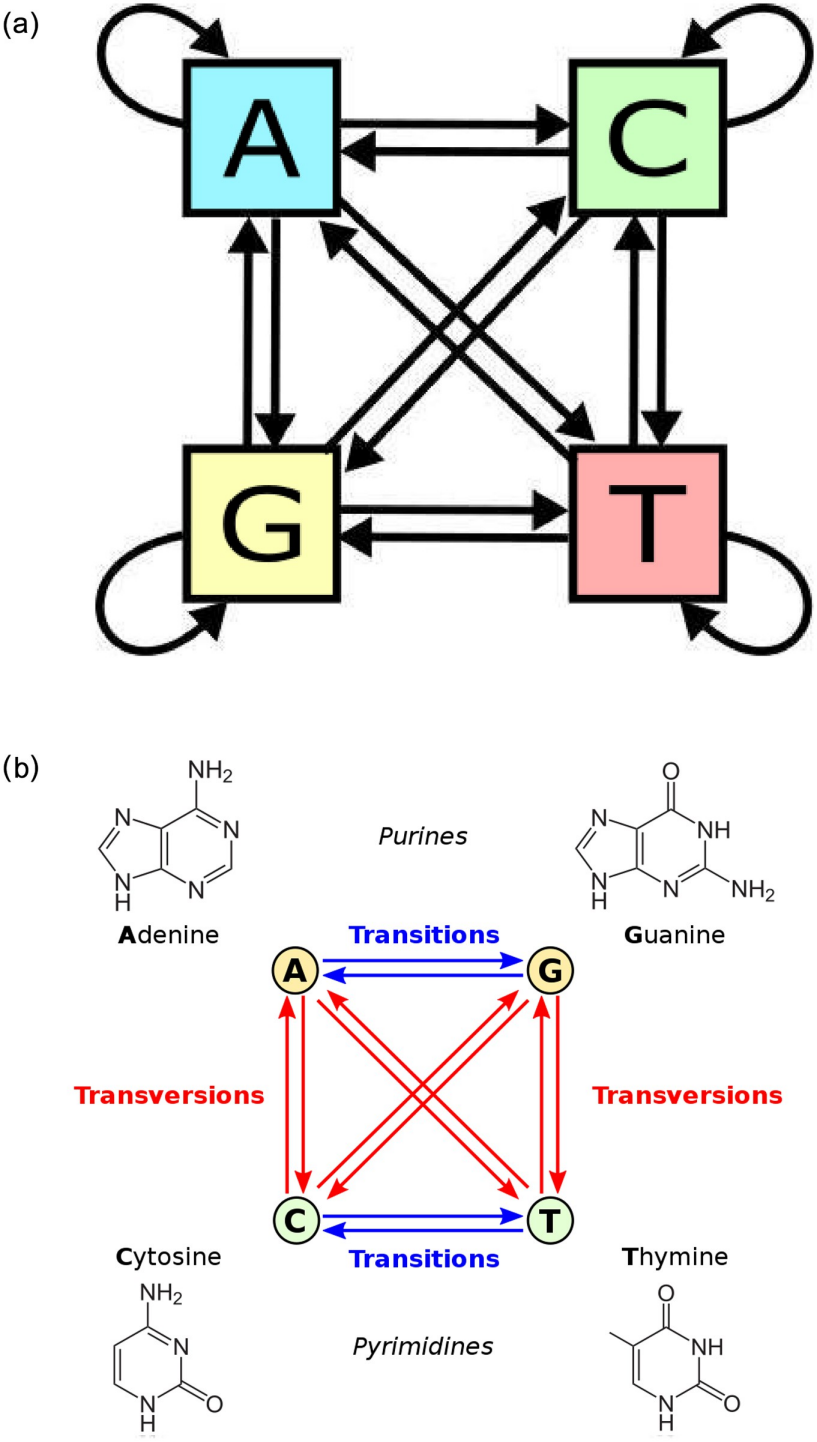
State transition diagrams. (a) Markov state transitions, (b) Corresponding nucleotide transversions and transitions.

This study proposes to model these random nucleotide mutations as a Markov Chain embedded Poisson process. Our method uses a Markov Chain Model to represent the nucleotide substitution process, whereby each nucleotide A, G, T, C (Adenine, Guanine, Thymine, Cytosine) is given 4 possible transition states in the system. By mapping each state in a state-transition diagram (Fig 3), we observe that these four states are recurrent and communicate with each other within a closed-loop chain. The transition rates between these states represent their rate of mutation which we assume to be a Poisson random process with arrival rate λ_*t*_, given by the probability mass function:

$$\begin{array}{c} P(x)=\frac{e^{-\lambda_{t}}\lambda_{t}^{x}}{x!} \end{array}$$ (1)

where the random variable x represents the number of occurrences and the parameter λ_*t*_, the Poisson rate coefficient. The overall resulting model is a positive-recurrent Continuous-Time Markov Chain (CTMC) embedded Poisson process (Fig 3).

Using this model, we can predict the time duration between mutations for each VOC based on their historical divergence rates from the parent strain. The Poisson arrival rate is then multiplied with the Markov transition probabilities between nucleotides to obtain the resulting 4x4 transition rate matrix whereby each element represents the number of times we can expect each specific nucleotide substitution to occur in any given month. This can help construct future evolution of each nucleotide location and hence construct future potential virus RNA sequences.

### 2.2 Spatial gene sequence analysis

**Fig 4:**
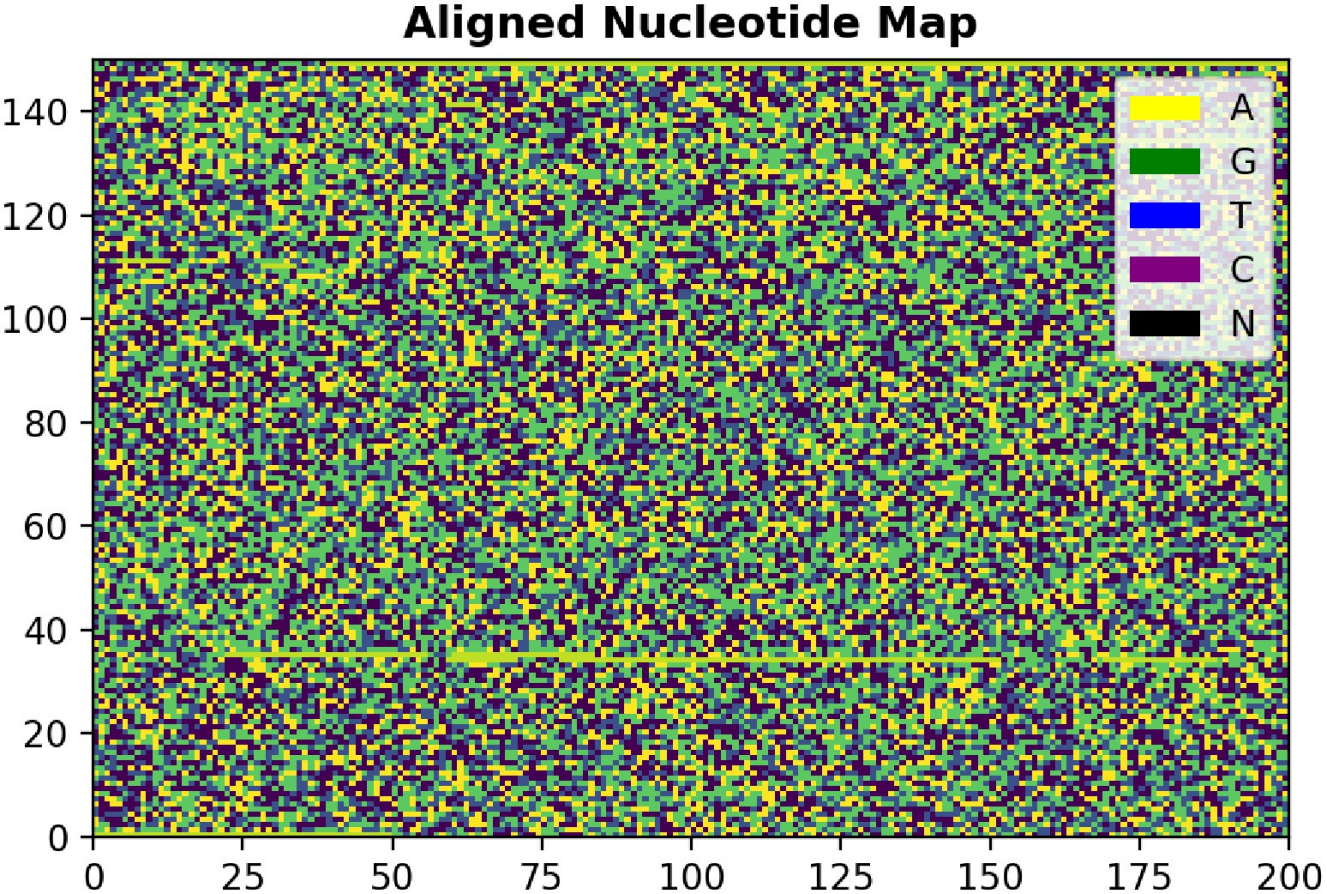
Sample nucleotide map.

Beyond the time-series analysis, we can also extend our sequence analysis into the spatial gene sequence domain. By taking the approximately 30,000-characters long nucleotide sequence and assigning them numerical values (A = 50, G = 100, T = 200, C = 250), we can convert the string into a numpy array. This allows us to easily plot out the genome sequence using the matplotlib pcolor method to create visual representation of the constituent nucleotides of the sequence. By comparing the array plots of different sample sequences, we can make quick comparisons on the different mutations along the genome, along with their corresponding index location on the RNA chain. The resulting visual plot is depicted in (Fig 4). By running the above spatial gene sequence analysis over many genome samples in the genome database, we can derive a spatial gene sequence representation of how the mutation rate varies over time for each nucleotide index. This spatial representation enables us to conduct characteristic analysis such as a heatmap of mutation points, from which we can derive the distribution of mutation interoccurrence distances.

As discovered in the previous study done by Muino et al [4], natural diseases appear to display a characteristic “footprint” pattern of localized somatic mutations, such as the Poisson distribution commonly found in existing literature, or the unique power law relationship in short-range cancer gene sequences. In this analysis, we apply a similar method to characterize the spatial gene sequence “footprint” of the SARS-CoV-2 virus.

In parallel with our time series model, we propose a Poisson model (with rate λ_*s*_) for the arrival process of mutations within a given spatial nucleotide sequence interval. Due to the memoryless property of Poisson processes this amounts to exponential distribution (with parameter λ_*s*_) for the nucleotides distances between two consecutive mutations. Plotting the logarithm of the number of occurrences of mutations versus the nucleotides distances between consecutive mutations, we expect a linear relationship if the exponential distributed interoccurrence distance (and equally Poisson distributed number of mutations) assumption is correct.

$$\begin{array}{c} P(x)=e^{-\lambda_{s}}\lambda_{s}^{x} \end{array}$$ (2)

$$\begin{array}{c} \text{log}(\lambda_{s})=\alpha +\beta x. \end{array}$$ (3)

Taking the logarithm of the exponential distribution probability density function given by Eq (2), we can represent the Poisson process in a linear form Eq (3) where λ_*s*_ represents the number of mutations in the gene sequence space, and *x* is a measure of the nucleotide distance between subsequent mutations along the gene sequence. This allows us to establish the linear relationship between the two variables in the log-scale, given by a gradient of *β* and corresponding intercept of *α*. Therefore, this relationship can be straightforwardly determined from the linearity of its log-linear histogram, which we will explore in (section 4) by performing linear regression in the logarithmic scale of nucleotide distances.

## 3 Data

**Fig 5:**
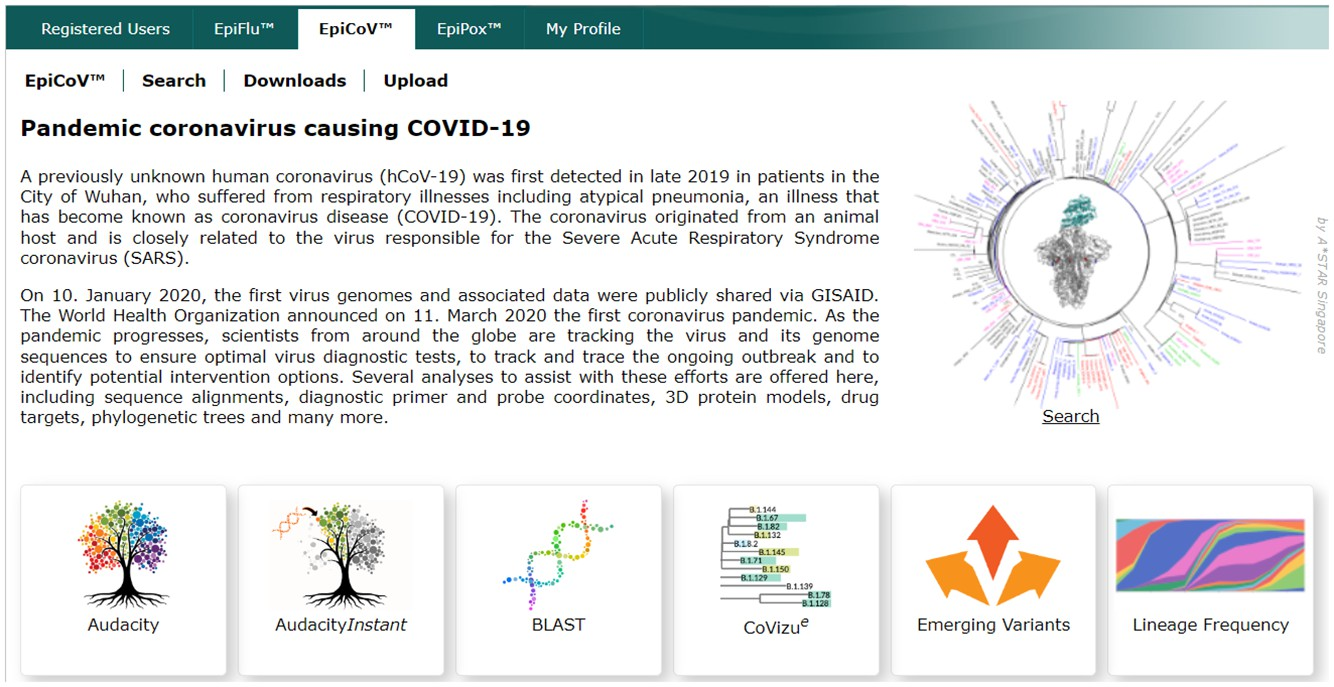
GISAID EpiCoV database [16].

This research accesses the GISAID EpiCoV database for SARS-CoV-2 genome sequences as the data source for training the model. GISAID [16] is a public data-sharing platform that aggregates virus genome submissions from around the world. The EpiCoV database includes sequence alignments, 3D protein models, drug targets and phylogenetic trees (Fig 5).

**Fig 6:**
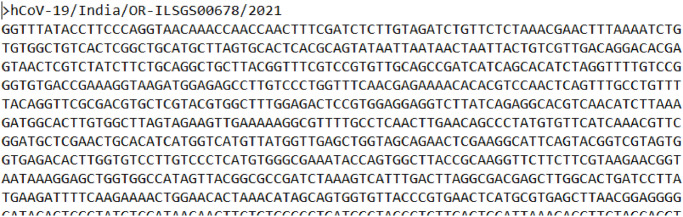
Nucleotide sequence (truncated).

The genomic sequences can be downloaded directly from the database as a zip folder consisting of two files, one with a format of *sequences.fasta* and another corresponding *metadata.tsv* file which contains S1 File such as the date of submission, originating lab, country, variant, and so on. The main information is obtained from the *sequences.fasta* file which encodes a string of approximately 30,000 characters of A, G, T, C corresponding to the nucleotides (Fig 6).

### 3.1 Data pre-processing

Before we can use the GISAID EpiCoV data for this study, we have to perform several data pre-processing steps to ensure that the sequences are structurally tractable. Firstly, the accession data is filtered for complete nucleotide genomes without any ‘Null’ entries along the sequence. Next, the samples are aligned via *Nucleotide BLAST* to account for any insertions or deletions in the sequence which could be problematic for substitution analysis due to frameshifted sequences. Finally, the filtered and aligned sequences are downloaded into a single *sequences.fasta* file for ingestion into our model. In this study, our models ingest SARS-CoV-2 sequence data from January to March 2022.

**Fig 7:**
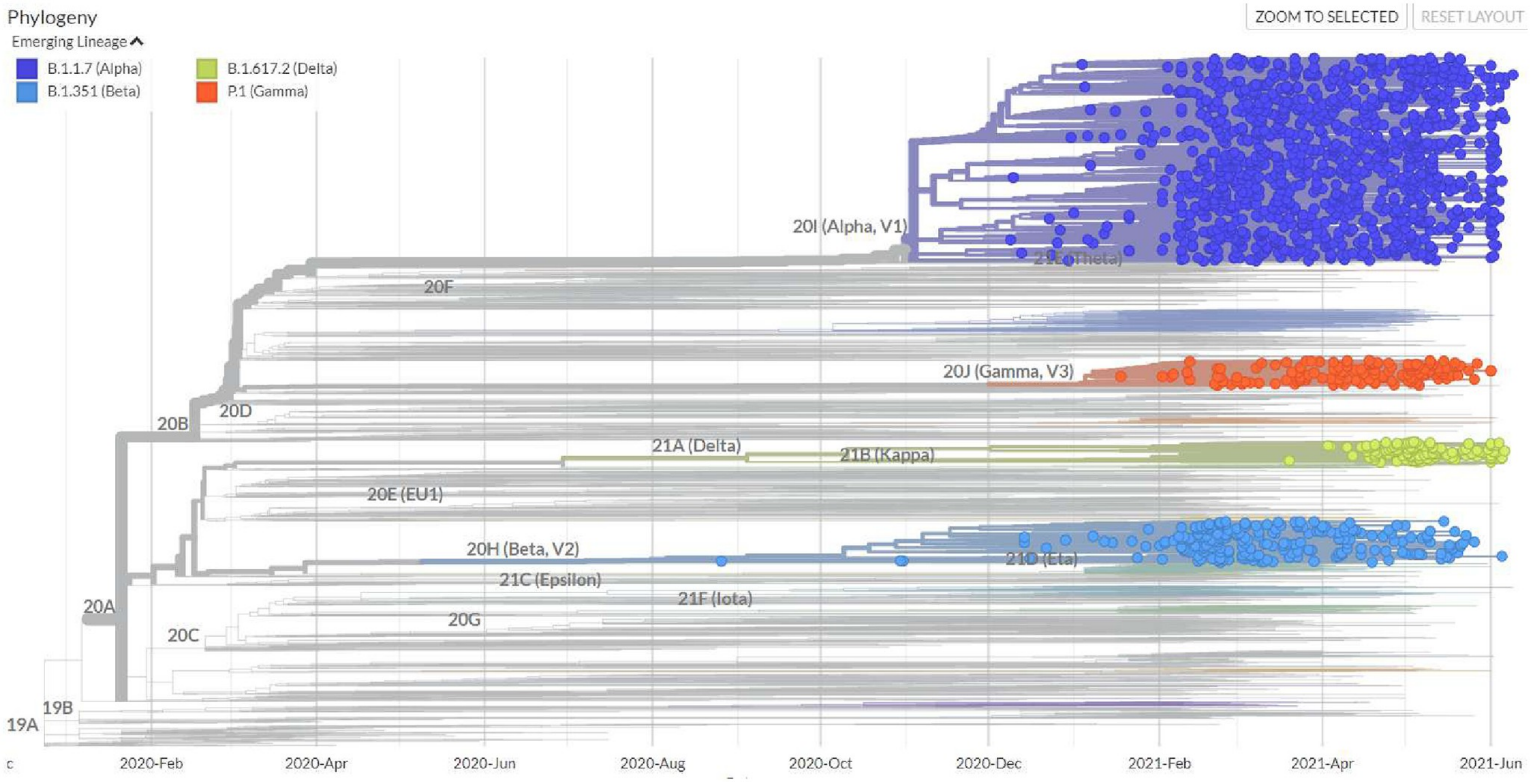
SARS-CoV-2 phylogenetic tree [17].

The database also quantifies the divergence of each submitted genome versus the reference strain, which is the original sequence first isolated from Wuhan on 01 Dec 2019. GISAID provides the definition of the *divergence* metric [16] as the number of changes (mutations) in the genome from the ancestor strain. Therefore, analyzing the SARS-CoV-2 Phylogenetic Tree (Fig 7) allows us to determine the divergence of each VOC upon first detection. Furthermore, by tracing the elapsed time from the original sequence to the emergence of each VOC, we can divide the elapsed time by their respective divergences in order to calculate their individual divergence rates in the time domain.

## 4 Results

### 4.1 Time series results

**Fig 8:**
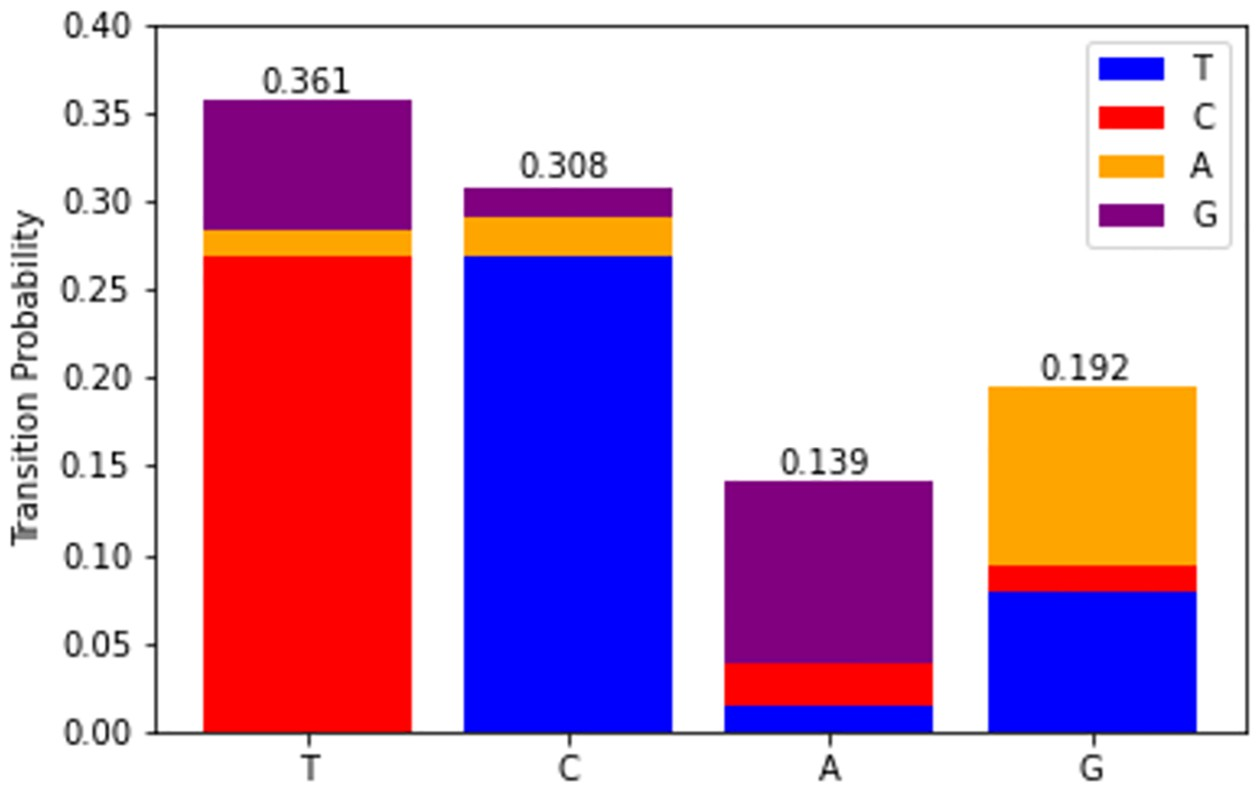
Markov transition probabilities.

GISAID’s empirical data with hundreds of fully sequenced genome submissions are ingested into the proposed model to obtain the transition probabilities each nucleotide mutation. The transition rate matrix can be visualized in a stacked bar chart whereby the x-axis represents each type of nucleotide and the stacked bars indicate their probability to be substituted by a corresponding nucleotide (Fig 8).

**Fig 9:**
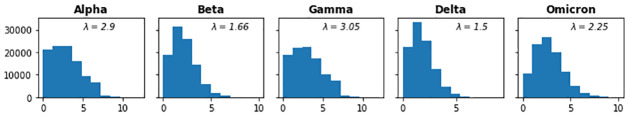
Poisson histograms of five key VOCs.

We also estimate the mutation rate of the next mutation that would occur for each VOC, where the divergence is given by a Poisson process for each of the four key variants. Their respective Poisson distributions are simulated over a large number of samples and then plotted as a histogram with corresponding mean mutation rates (λ_*t*_) which represents the number of expected nucleotide mutations per month for each VOC (Fig 9).

**Fig 10:**
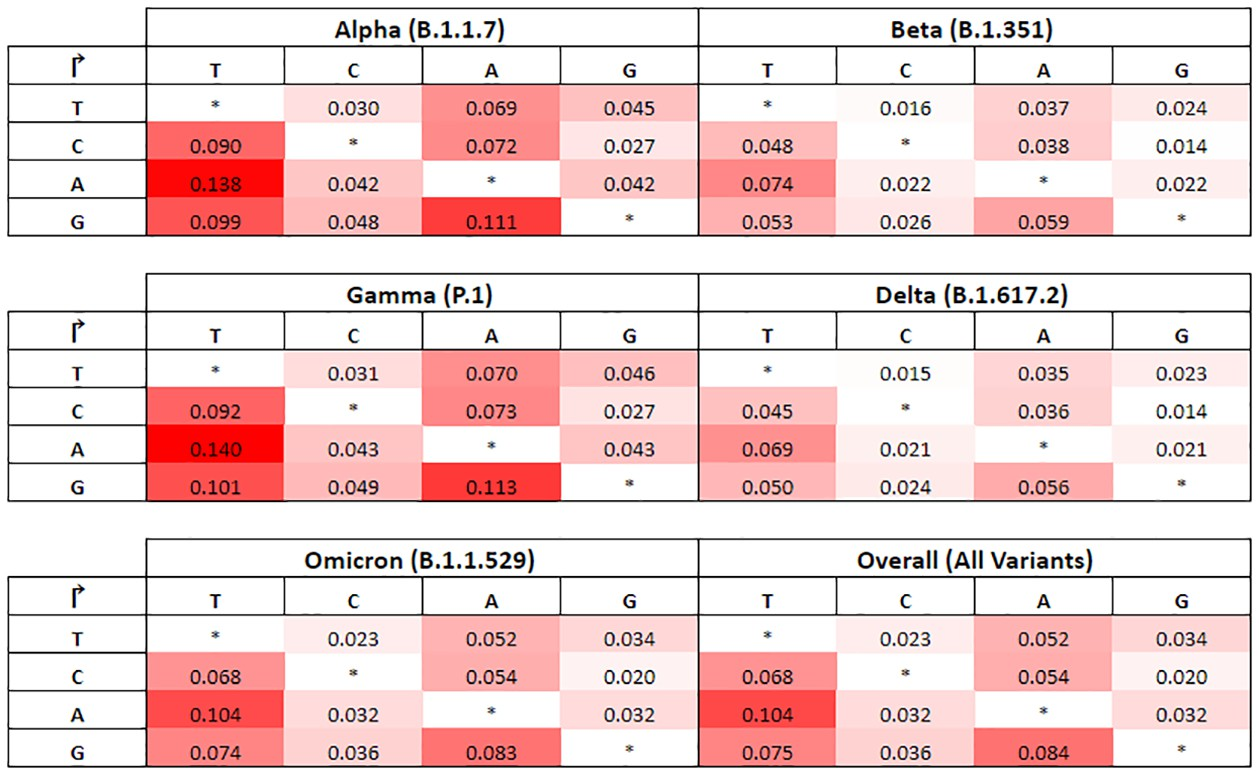
4x4 mutation rate matrices.

Multiplying the Markov transition probabilities (Fig 8) with the Poisson rate coefficients (Fig 9) will give us the specific mutation rate of each type of nucleotide substitution for each strain. The resulting mutation rate matrix is tabulated in (Fig 10) for each of the five key VOCs, whereby each value represents the mean rate of mutation per month for each nucleotide substitution from row to column. Cells with larger values are visually represented with darker colour shading.

The overall calculated mutation rate also has a Poisson mean of λ_*t*_ = 2.27 mutations per month, which closely matches the average of 2 mutations per month as cited from empirical results in the existing literature [18]. We validate the statistical robustness of our results by conducting a one-sample t-test on the sample mean compared with the expected population mean. The obtained test statistic T = 1.038, which falls within the 95% region of acceptance: [-1.984, 1.984] and a 95% confidence interval of [1.754, 2.786]. These results show that our findings display statistical similarity to previously discovered literature values [9, 18].

### 4.2 Spatial gene sequence results

**Fig 11:**
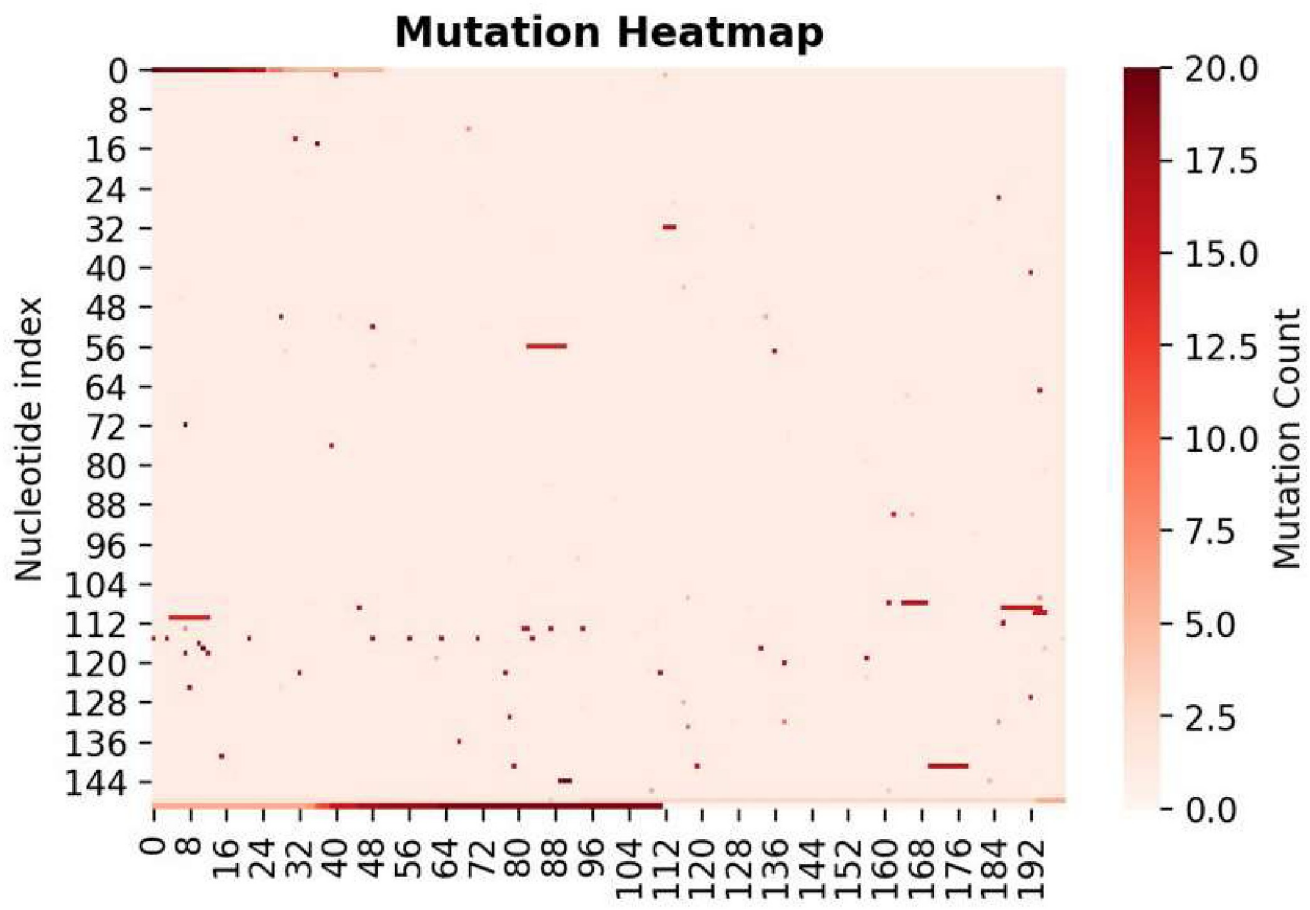
Mutation heatmap.

By making genomic comparisons of the 150x200 nucleotide maps across multiple sequences, we can map each point mutation occurrence on the mutation array. A cumulative count at each index allows us to plot a heatmap of mutation hot spots along the genome (Fig 11).

**Fig 12:**
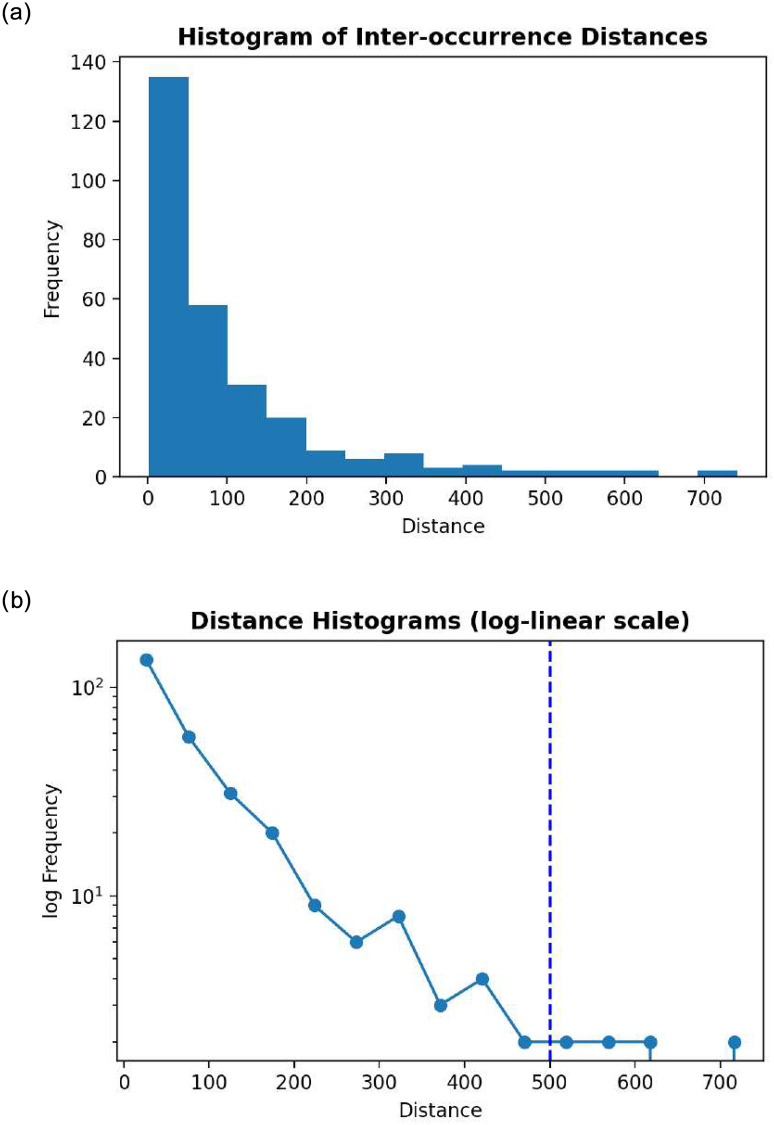
Interoccurrence distance histograms. a) Linear, b) Log-linear.

Taking the spatial gene sequence distance between indices of consecutive mutations along the genome, we can then calculate the mutation interoccurrence distances. Plotting these interoccurrence distances as a histogram on a linear (Fig 12a) and log-linear scale (Fig 12b) allows us to observe their spatial gene sequence distribution, given by a Poisson mean of approximately λ_*s*_ = 102 spatial nucleotide distance between mutations. To validate these results, we further conducted a linear regression test to verify the log-linearity of short range mutation distances. This test yielded a coefficient *R*^2^ = 0.708, which indicates that there exists the presence of a linear relationship in the logarithmic spatial domain.

## 5 Discussion

A detailed look at both our time-series and spatial gene sequence results reveals some interesting preliminary insights to the phylodynamics of the SARS-CoV-2 virus. However, we also note that there are several limitations to this simple substitution model that can be improved in future work.

### 5.1 Mutation rates differ across strains

A comparison of the mutation rates between the 5 key VOCs (Fig 9) indicates that the mutation rates for each key VOC differs slightly. The Alpha and Gamma variants are displaying a faster rate of evolution with Poisson coefficients of 2.90 and 3.05 mutations per month respectively, whereas the Beta and Delta strains are mutating at about half the rate with arrival rate of 1.66 and 1.50, respectively. A possible interpretation of this finding is that the Beta and Delta variants are more stable in nature due to certain genetic advantages over its competitors. This result also corroborates existing medical findings which suggest that each strain exhibits unique virus characteristics, especially in the gene expressions of their spike protein, which causes them to have different binding affinity to the human ACE-2 receptors ultimately resulting in varied mutation dynamics [18].

### 5.2 Mutation rates differ by nucleotides

From the Markov state transition probabilities (Fig 8), it is worth noting that in general, the nucleotides T (Thymine) and C (Cytosine) have higher propensities to be substituted in the chain, with almost twice the probability over A (Adenine) and G (Guanine). This interesting property is observed across all variants in (Fig 10) where we can observe clear asymmetry in the mutation rates, notably that pyrimidine transitions occur more often than purine transitions, while transversions between the two different kinds of nucleotides are infrequent by contrast (Fig 3). This could point to evidence that there exists some evolutionary selection mechanism for mutation types as verified by De Maio (2021) who mapped the selection ratios for different mutation types [7]. A plausible biological explanation could be the predominance of certain nucleotides found in the spike protein receptor binding domain (RBD) where most of the mutations have been detected to occur with higher frequencies (Fig 2), possibly due to selective escape from immune response [19].

### 5.3 Exponentially distributed interoccurrence distances

From the histogram of interoccurrence distances (Fig 12a), we observe that the distances appear to be decreasing exponentially, suggesting that the distribution of number of mutations in the gene sequence space is Poisson in nature. To verify this using our linearized model in Eq 3, we check the linearity of the log-linear histogram and note that at short-range distances (< 500 nucleotides), there indeed exists a linear relationship between frequency and distance. A similar trend is observed for all 5 VOCs, therefore we present the histogram for the Omicron variant as an example to visualize the corresponding distribution.

This finding draws some interesting parallels with the cancer mutation research by Muiño et al [4], where they discovered that cancer genomes exhibit power-law interoccurrence distances in its short-range sequences, whereas our study suggests a Poisson mutation occurrence signature with exponentially distributed interoccurrence distances. This result is of particular interest since it could represent a characteristic “footprint”of the spatial gene sequence pattern underlying the SARS-CoV-2 mutation mechanism, much like the “Kataegis” phenomenon that was noted by the cancer study.

The significance of identifying the spatial Poisson behaviour of mutations in the gene sequence enables the ability to predict the approximate location along the genome where we can expect the next mutation to occur. This could allow virologists to focus their research on the potential impacts of future mutation positions in the short range. Therefore, a potential application of this finding could be to contribute statistical information for the timely or even predictive update of vaccine sequences in anticipation of the next VOC emergence, which is very complex and open research question that no existing models have been able to achieve with sufficient accuracy and confidence as of yet.

### 5.4 Limitations

There are several limitations of this model, with several constraints pertaining to the dataset available from the GISAID database, while some limitations are inherent to the underlying steady-state assumptions behind the analysis method.

#### 5.4.1 Data skewness

Firstly, it has been highlighted that the data points are heavily skewed toward countries with a higher number of genome submissions, especially from first world countries such as the UK and US with better technology infrastructure and more laboratories that are able to collect and sequence the genomes of the virus samples. Secondly, it is also established by several other collaborators of GISAID that a large proportion of the submitted reports could be biased towards reported ‘bad’ strains only, as benign samples would be omitted due to the lack of manifestation of the COVID-19 disease [20].

#### 5.4.2 Steady-state assumption

Another limitation to the analysis method is the inherent assumption of a steady-state behaviour being exhibited by the virus. By assuming a Poissonian and Markovian nature of virus mutations, our methods analyze the mutation rate as a distribution centered around a fixed mean and standard deviation. However, in reality the different strains of the virus could mutate at different rates at various time points in the outbreak. This could be due to a variety of external factors that affect its replication rate, such as the population of infected individuals. In essence, our study only captures a “snapshot” in time of the current phylodynamic state of the virus, but does not account for future changes in its characteristics. Further studies by moving time or genome sequence location windows can reveal non-homogeneous Poisson characteristics.

#### 5.4.3 Indel mutations

Apart from the limitations of the dataset, there are also several limitations in the proposed model in this paper. This preliminary model that we have built currently only considers the mechanism of nucleotide substitution. However, in reality there are other mutation mechanisms such as indels (the insertion or deletion of nucleotides) in the virus genome, for which this rudimentary substitution model does not account for. Furthermore, the proposed model is focused on the arrival times of mutations, but does not contain any information on the location of the mutagenic event along the virus genome.

#### 5.4.4 Viral impact analysis

Lastly, although we are able to provide a prediction of the Poisson mutation rate with its corresponding Markov nucleotide transition, the prediction result bears no information on the impact of the mutation on the SARS-CoV-2 virus characteristics. As such, further consultation with virologists is required to assess the impact of the predicted mutation to ascertain whether the change is positive or negative in terms of the realizations of the COVID-19 disease it causes [21]. Alternatively, we can explore some parametric stochastic models in order to predict the emergence of VOCs by analyzing historical phylodynamic data from the GISAID database.

## 6 Conclusions

Our study presents a simple time-series model to estimate the mutation rate of the SARS-CoV-2 virus as a Markov Chain embedded Poisson process. The overall calculated Poisson mutation rate is λ_*t*_ = 2.27 mutations per month, which closely matches the findings from existing literature [19]. We can thus expect a new mutation to occur with inter-arrival time given by an exponential distribution with a mean of every 13 days. We found that the mutation rates for each key VOC differs slightly, further noting that the pyrimidine nucleotides Thymine and Cytosine have higher propensities to be substituted.

In the spatial gene sequence model, we calculated the interoccurrence distances between nucleotide mutations to have an exponentially distributed mean of λ_*s*_ = 102 nucleotide distance, signifying a Poisson mutation occurrence pattern in the spatial domain. We also heatmapped the interoccurrence of mutations along the genome, observing a log-linear relationship between mutation distance and frequency in short-range nucleotide distances. This finding draws significant parallels with the cancer mutation research by Muiño et al [4], being of particular interest since it could be representing a part of a characteristic spatial gene sequence pattern underlying the SARS-CoV-2 mutation mechanism. To our knowledge, we are the first study to have characterized a pattern of interoccurrence distances between nucleotide mutations in the SARS-CoV-2 genome. The contributions of this research could potentially pave the way for future work, such as applying more advanced spatial pattern recognition algorithms in order to characterize other different kinds of virus families.

As the SARS-CoV-2 virus continues to mutate, not only will the 5 dominant VOCs continue to descendant strains, there could be even more independent variants emerging. Many of these variants are classified as variants of interest (VOIs) and are under careful investigation by WHO due to their potential to become a VOC in the future [22]. As such, in the future we hope to develop a suitable mutation model which can be applied to the descendants of the 4 VOCs, or onto the list of VOIs in order to understand their phylodynamics before they potentially mutate into a more dangerous strain. As of this writing, the WHO has been investigating two Omicron subvariant (BA.1, BA.2), which have shown “greater immune escape properties and higher transmissibility”, threatening to unleash a new wave of infections around the world [23]. Thus, the fortuitous timing of this work could contribute to the analysis of new variants and subvariants in the ongoing fight against the COVID-19 pandemic.

## Supporting information

S1 File (ZIP)
