# Supplementary material for: Modeling SARS-CoV-2 nucleotide mutations as a stochastic process
Source: PLoS One. 2023 Apr 28;18(4):e0284874. doi: 10.1371/journal.pone.0284874 (PMC10146438; doi:10.1371/journal.pone.0284874)
Supplement: S1 File — (ZIP) [file pone.0284874.s001.zip › image14.pdf]

Substitution Models	Parameters	Model Property	Transition Matrix
**1. JC69** (Jukes & Cantor, 1969)	1 (μ)	Assumes equal mutation rates (μ)	$\begin{pmatrix} * & \frac{\mu}{4} & \frac{\mu}{4} & \frac{\mu}{4} \\ \frac{\mu}{4} & * & \frac{\mu}{4} & \frac{\mu}{4} \\ \frac{\mu}{4} & \frac{\mu}{4} & * & \frac{\mu}{4} \\ \frac{\mu}{4} & \frac{\mu}{4} & \frac{\mu}{4} & * \end{pmatrix}$
**2. K3P** (Kimura, 1980)	3 (α, β, γ)	Assumes unique rate for transitions, (α) and 2 types of transversions, (β, γ)	$\begin{pmatrix} * & \alpha & \beta & \gamma \\ \alpha & * & \gamma & \beta \\ \beta & \gamma & * & \alpha \\ \gamma & \beta & \alpha & * \end{pmatrix}$
**3. F81** (Felsenstein, 1981)	4 ($\pi_A, \pi_C, \pi_G, \pi_T$)	Assumes unique rate for each of the bases ($\pi_A, \pi_C, \pi_G, \pi_T$)	$\begin{pmatrix} * & \pi_G & \pi_C & \pi_T \\ \pi_A & * & \pi_C & \pi_T \\ \pi_A & \pi_G & * & \pi_T \\ \pi_A & \pi_G & \pi_C & * \end{pmatrix}$
**4. CTMC embedded Poisson** (proposed method)	12 (λ_{ij})	Assumes unique Poisson rates for each specific base substitution (λ_{ij})	$\begin{pmatrix} * & \lambda_{GA} & \lambda_{CA} & \lambda_{TA} \\ \lambda_{AG} & * & \lambda_{CG} & \lambda_{TG} \\ \lambda_{AC} & \lambda_{GC} & * & \lambda_{TC} \\ \lambda_{AT} & \lambda_{GT} & \lambda_{CT} & * \end{pmatrix}$
