# Supplementary material for: Modeling SARS-CoV-2 nucleotide mutations as a stochastic process
Source: PLoS One. 2023 Apr 28;18(4):e0284874. doi: 10.1371/journal.pone.0284874 (PMC10146438; doi:10.1371/journal.pone.0284874)

Current S-Model

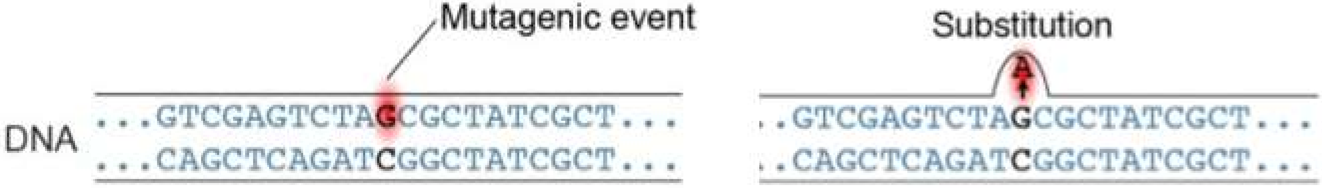

Extended SID-Model

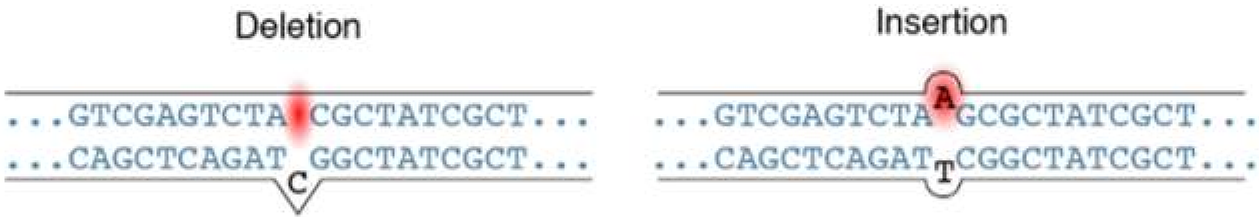

- Predict the expected **location** of the mutation
- Predict **how often** these mutations occur
- Predict the corresponding **Substitution, Insertion,** or **Deletion** of nucleotides

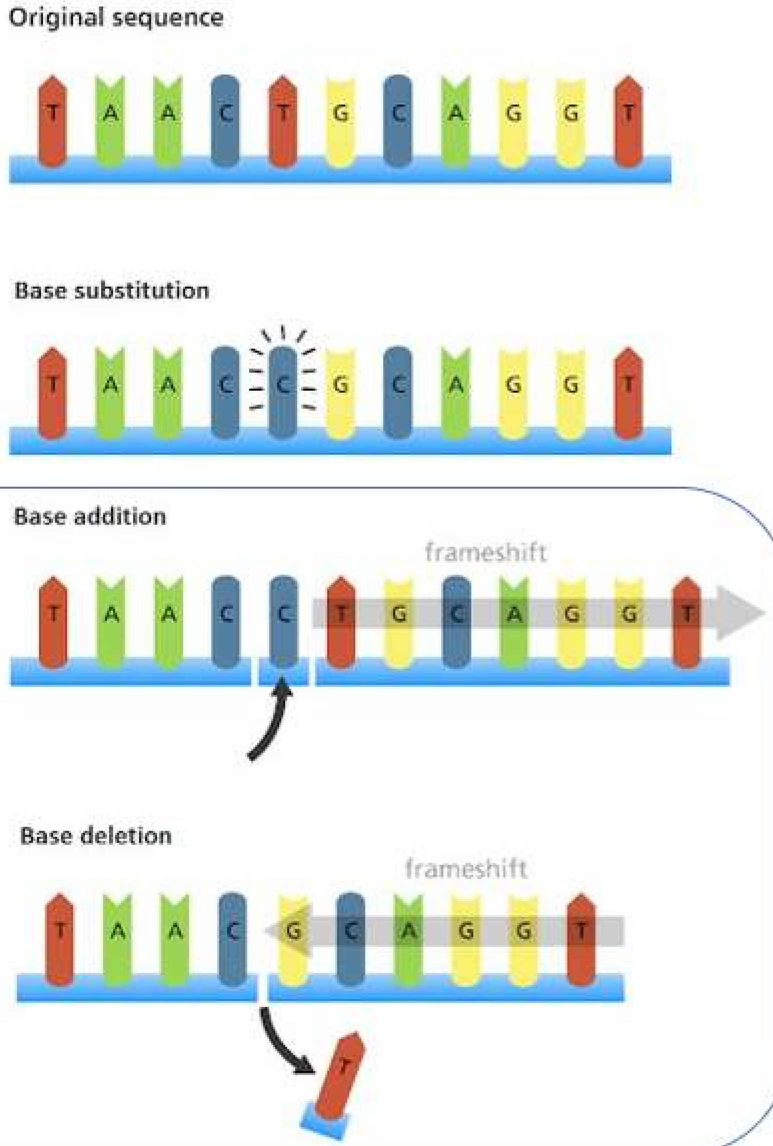

Supplement: S1 File — (ZIP) [file pone.0284874.s001.zip › image17.pdf]
