# Supplementary material for: Modeling SARS-CoV-2 nucleotide mutations as a stochastic process
Source: PLoS One. 2023 Apr 28;18(4):e0284874. doi: 10.1371/journal.pone.0284874 (PMC10146438; doi:10.1371/journal.pone.0284874)
Supplement: S1 File — (ZIP) [file pone.0284874.s001.zip › image18.pdf]

RESEARCH ITEM	2021				2022	
**Chapter 1: Mutation Models** (Parametric approach)	Q1	Q2	Q3	Q4	Q1	Q2
a. Literature Review	**a. Literature Review**					
b. Time-Series Model		**b. Time-Series Model**				
c. Spatial Model			**c. Spatial Model**			
d. Time-Space Model				**d. Time-Space Model**		
**Publication #1:** *Journal of Theoretical Biology, Bioinformatics*					**Publication #1**	
e. Non-homogenous Model (Time-Series Model extension)					**e. Non-Homogenous**	
f. Indels Model (Spatial Model extension)					**f. Indels Model**	

RESEARCH ITEM	2022		2023			
**Chapter 2: VOC/VOI Models** (Data-driven approach)	Q3	Q4	Q1	Q2	Q3	Q4
a. Literature Review	**a. Literature Review**					
b. Codon Model		**b. Codon Model**				
c. LSTM RNN Model			**c. LSTM RNN Model**			
d. VOC/VOI Prediction Model				**d. VOC/VOI Prediction Model**		
**Publication #2**: *Royal Society, Interface Focus*					**Publication #2**	
e. Transfer Learning Model					**e. Transfer Learning Model**	
f. Geographical Analysis					**f. Geographical Analysis**	
