# Supplementary material for: Modeling SARS-CoV-2 nucleotide mutations as a stochastic process
Source: PLoS One. 2023 Apr 28;18(4):e0284874. doi: 10.1371/journal.pone.0284874 (PMC10146438; doi:10.1371/journal.pone.0284874)
Supplement: S1 File — (ZIP) [file pone.0284874.s001.zip › image1a.pdf]

$$recall = \frac{true\ positives}{true\ positives\ \ + false\ negatives} \qquad precision = \frac{true\ positives}{true\ positives + false\ positives}$$
