# Supplementary material for: Modeling SARS-CoV-2 nucleotide mutations as a stochastic process
Source: PLoS One. 2023 Apr 28;18(4):e0284874. doi: 10.1371/journal.pone.0284874 (PMC10146438; doi:10.1371/journal.pone.0284874)
Supplement: S1 File — (ZIP) [file pone.0284874.s001.zip › image5.pdf]

Needleman-Wunsch

match = 1 mismatch = -1 gap = -1

		G	C	A	T	G	C	G
	0	-1	-2	-3	-4	-5	-6	-7
**G**	-1	1	0	-1	-2	-3	-4	-5
**A**	-2	0	0	1	0	-1	-2	-3
**T**	-3	-1	-1	0	2	1	0	-1
**T**	-4	-2	-2	-1	1	1	0	-1
**A**	-5	-3	-3	-1	0	0	0	-1
**C**	-6	-4	-2	-2	-1	-1	1	0
**A**	-7	-5	-3	-1	-2	-2	0	0
