# Supplementary material for: Modeling SARS-CoV-2 nucleotide mutations as a stochastic process
Source: PLoS One. 2023 Apr 28;18(4):e0284874. doi: 10.1371/journal.pone.0284874 (PMC10146438; doi:10.1371/journal.pone.0284874)
Supplement: S1 File — (ZIP) [file pone.0284874.s001.zip › imagec.pdf]

$$L = -y * \log(p) - (1-y) * \log(1-p) = \begin{cases} -\log(1-p), & if\ y = 0 \\ -\log(p), & if\ y = 1 \end{cases}$$
