# Supplementary material for: Modeling SARS-CoV-2 nucleotide mutations as a stochastic process
Source: PLoS One. 2023 Apr 28;18(4):e0284874. doi: 10.1371/journal.pone.0284874 (PMC10146438; doi:10.1371/journal.pone.0284874)
Supplement: S1 File — (ZIP) [file pone.0284874.s001.zip › imaged.pdf]

$$\mathrm{MCC} = \frac{TP \times TN - FP \times FN}{\sqrt{(TP + FP)(TP + FN)(TN + FP)(TN + FN)}}$$
