# Supplementary figures and images for: Modeling SARS-CoV-2 nucleotide mutations as a stochastic process
Source: PLoS One. 2023 Apr 28;18(4):e0284874. doi: 10.1371/journal.pone.0284874 (PMC10146438; doi:10.1371/journal.pone.0284874)

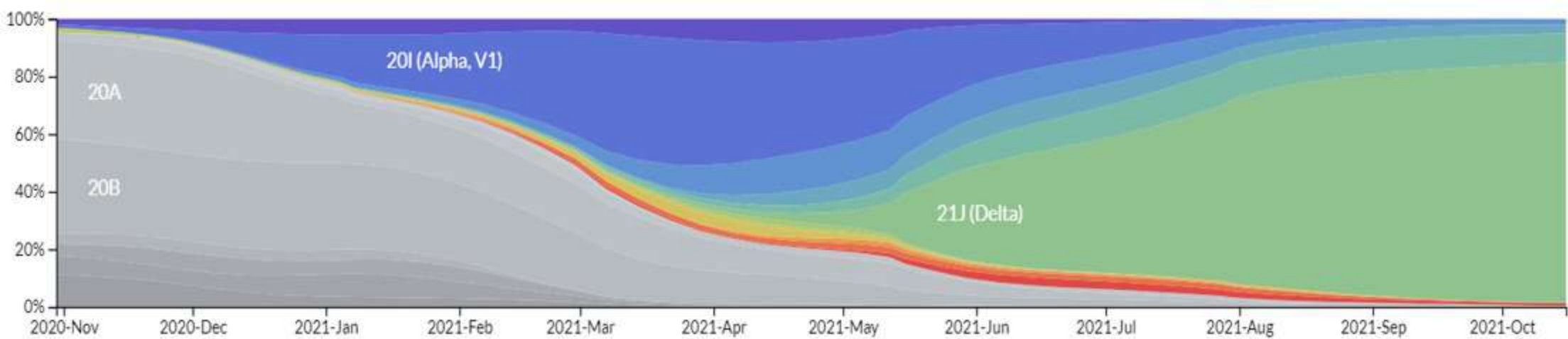

Supplement: S1 File — (ZIP) [file pone.0284874.s001.zip › image2.pdf]

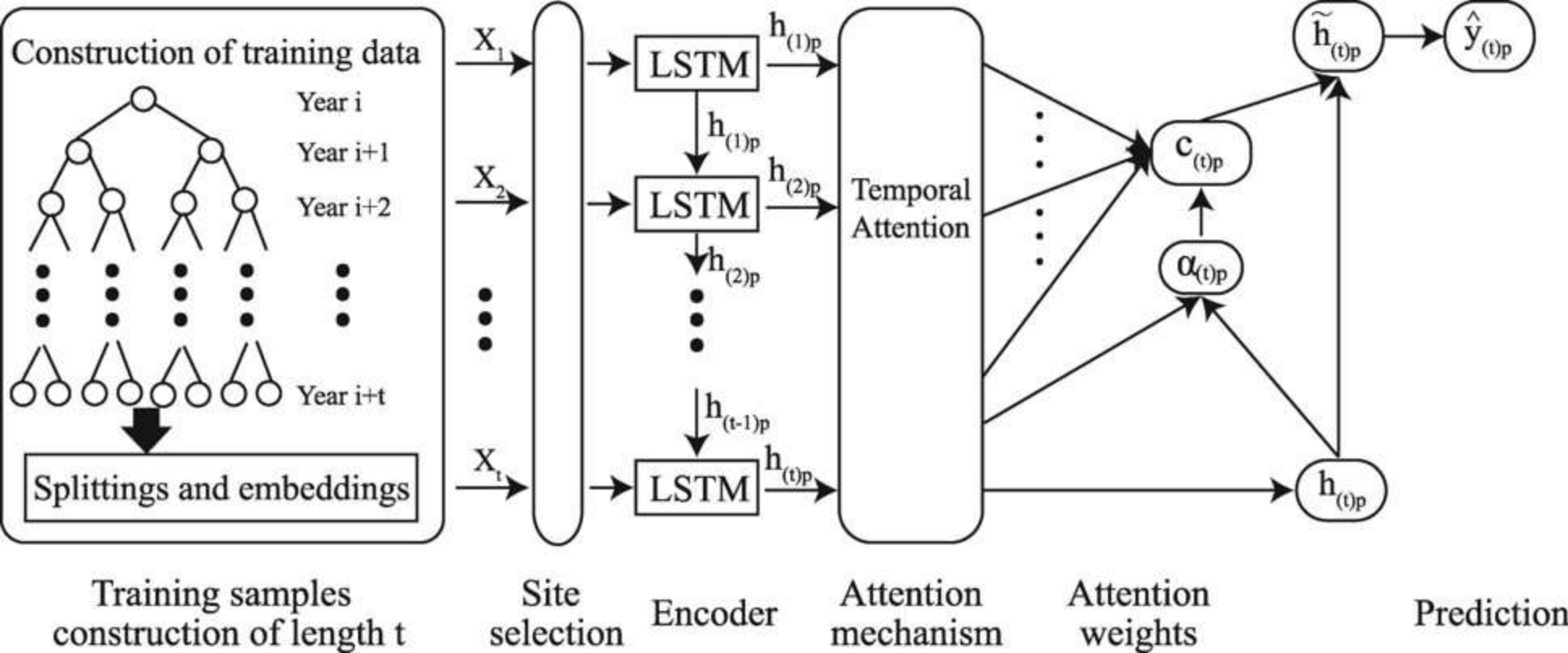

Supplement: S1 File — (ZIP) [file pone.0284874.s001.zip › image4.pdf]

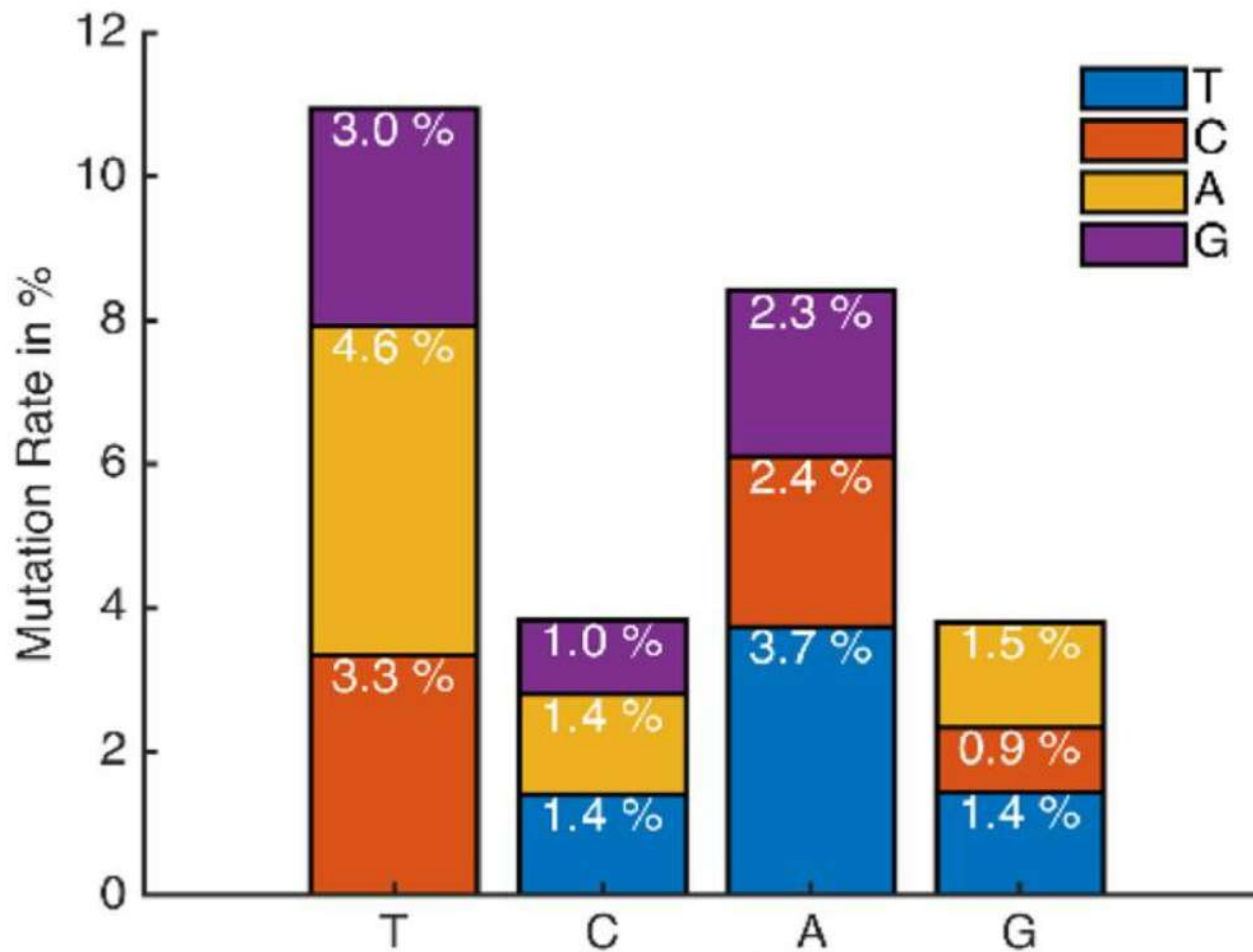

Supplement: S1 File — (ZIP) [file pone.0284874.s001.zip › image8.pdf]

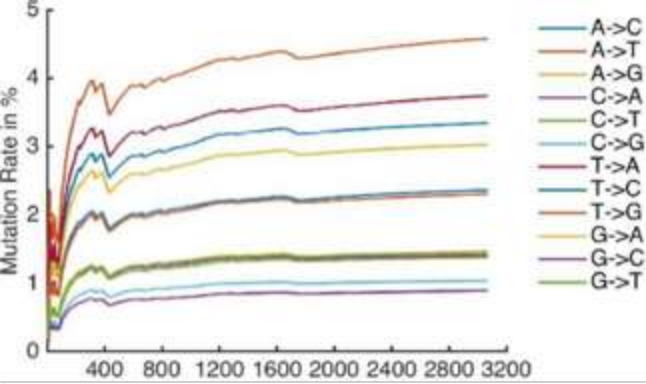

Supplement: S1 File — (ZIP) [file pone.0284874.s001.zip › image9.pdf]

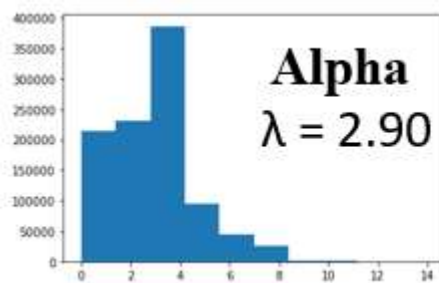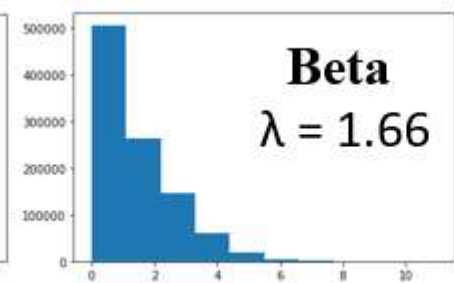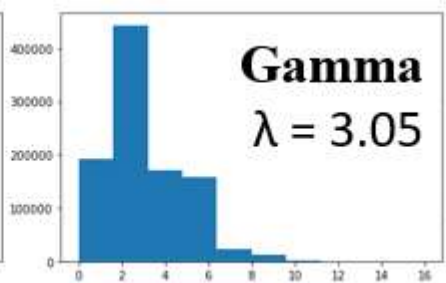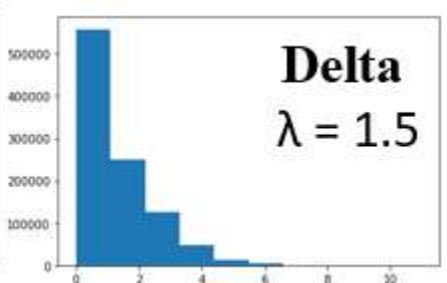

Supplement: S1 File — (ZIP) [file pone.0284874.s001.zip › imagea.pdf]

# Mutation Heatmap

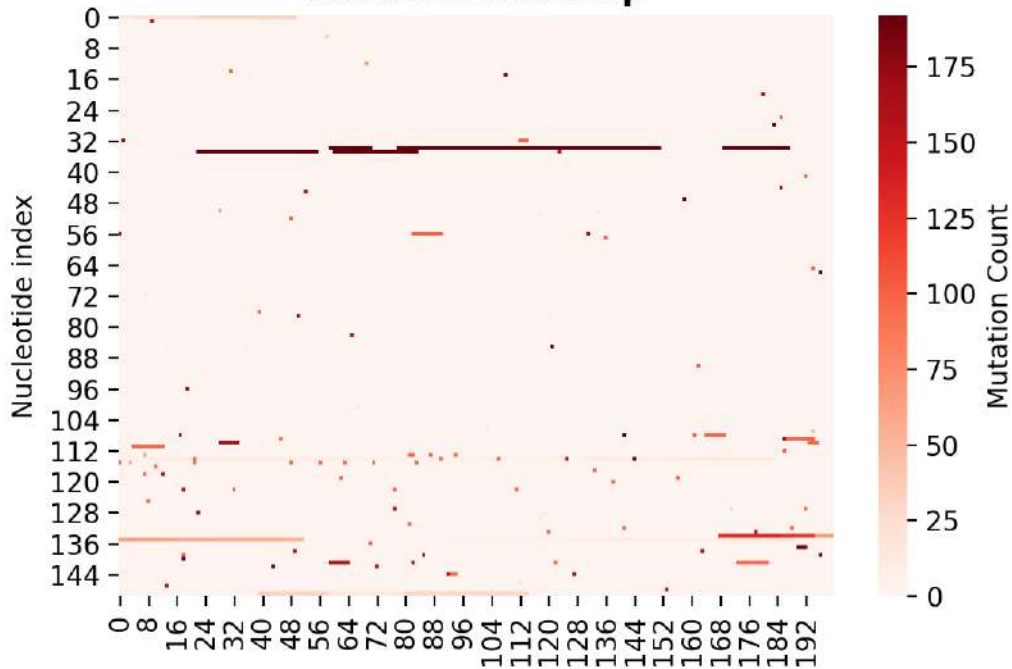

Supplement: S1 File — (ZIP) [file pone.0284874.s001.zip › Mutation Heatmap.pdf]

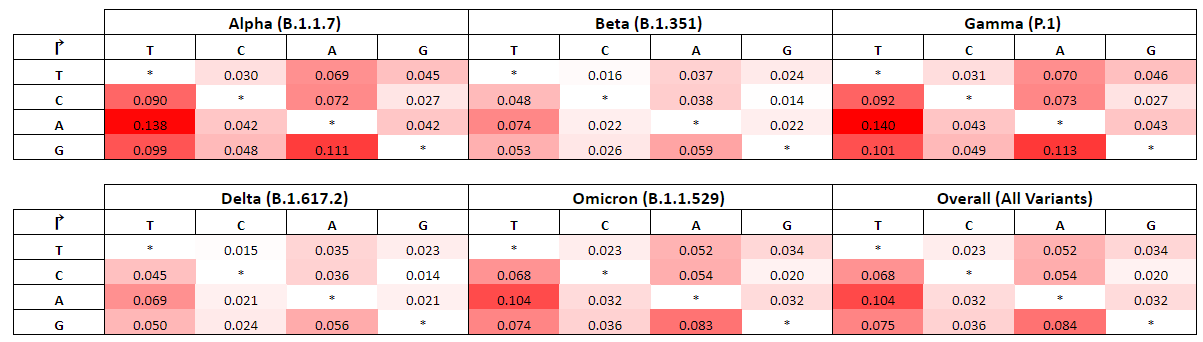

Supplement: S1 File — (ZIP) [file pone.0284874.s001.zip › Mutation Rate Matrix Heatmap.png]
